# Exploring the Relationship Between Entrepreneurship and Psychological Characteristics, and Corporate Social Responsibility Under Marketization

**DOI:** 10.3389/fpsyg.2021.693644

**Published:** 2021-07-28

**Authors:** Zhengshun Shen, Huaibin Li, Yancai Zhang

**Affiliations:** ^1^School of Economics and Management, Huaiyin Normal University, Huai'an, China; ^2^School of Business Administration, Dongbei University of Finance and Economics, Dalian, China

**Keywords:** the perspective of marketing, entrepreneurship, psychological characteristics, corporate social responsibility, marketization process

## Abstract

The present work aims to explore how and to what extent ventures can bear social responsibilities due to their increasingly important role in social and economic activities. Here, the correlation between entrepreneurship and psychological characteristics of entrepreneurs, and corporate social responsibility is analyzed from the perspective of entrepreneurial entrepreneurs based on marketization theories of enterprise activities. The β value of entrepreneurship in corporate social responsibility is obtained as 0.863 (*p* < 0.001) through a questionnaire survey (QS) and statistical analysis. It indicates that entrepreneurship has a significant positive impact on corporate social responsibility. The regression coefficient of the marketization process is 0.574, and the significance is <0.01. The results show that there is a significant positive correlation between the marketization process and corporate social responsibility at the level of 1%. When the marketization process is introduced, entrepreneurship significantly and positively impacts corporate social responsibility (β = 0.524, *p* < 0.001), while the marketization process has a great positive impact on corporate social responsibility (β = 0.502, *p* < 0.001). However, the coefficient of entrepreneurship and psychological characteristics decreases from 0.863 to 0.524. It reveals that its impact on corporate social responsibility is weakened by the introduction of the marketization intermediary variable, but the overall variation that the model can explain increases to 84.3%. The results show that the marketization process has a significant positive mediating effect on the relationship between entrepreneurship and corporate social responsibility, and entrepreneurship plays a very important role in corporate social responsibility. The content and methods are complete and comprehensive, and the results can provide scientific and effective references for subsequent studies.

## Introduction

Excellent enterprise leaders, especially, the new generation of entrepreneurial entrepreneurs, consciously shoulder the new mission entrusted by the new era and become the main force of China's new economic development, transformation, and upgrading (Windasari et al., 2020). This is not only a developmental and creational process for new companies but also their ingenious development of new businesses and new organizations. The entrepreneur economy is changing the government and society, and the view toward SMEs (Small and Medium-sized Enterprises) development is also changing (Mahto and McDowell, 2018). With the advent of the information age, issues related to corporate social responsibility have been widely reported by various media, and all sectors of society are concerned about whether corporate social responsibility is fulfilled (Wu and Song, 2019). Meanwhile, corporate social responsibilities vary among companies, affecting the performance of corporate social responsibility, which needs in-depth analysis (Vuong and Napier, 2015; Niu et al., 2021). Companies should not regard social responsibility as a burden that hinders their development. Instead, companies should actively undertake corporate social responsibility, which can coordinate the relationship between the company and other social stakeholders and stabilize the relationship among the company, the government, and the community. Meantime, the company's image and reputation can be upgraded, increasing social resources to promote company development and improve customer trust and social recognition (Olaniyi Banwo, 2020).

Entrepreneurial entrepreneurs are the promoters and implementers of entrepreneurial activities and play a vital role in enterprise development. Entrepreneurs can more effectively seek opportunities, organize resources, research strategies, and ultimately create new business processes and production units (Tangtenglam, 2020). Thus, entrepreneurs can develop the economy, realize their vision, and exert their potential (Qian et al., 2018). Research shows that entrepreneurship and entrepreneurial personal traits are crucial to the development of ventures (Papagiannis, 2018). The psychological characteristics of entrepreneurs are the key to entrepreneurial activities (Huang et al., 2021). Entrepreneurs often manage more resources and have a deeper influence than common employees, so their spiritual characteristics can affect the overall development of the enterprise and even society. At present, with the surge of private economy and venture enterprises, the influence of entrepreneurs' characteristics is expanding both in breadth and depth. Most previous studies lack research on the impact of entrepreneurs' characteristics on enterprises and society. Particularly, there is no detailed and modeled analysis of the entrepreneurs' characteristics. Therefore, from the perspective of marketization, the impact of entrepreneurs' characteristics should be paid much attention to.

Based on the research of domestic and international scholars, the spirit and psychological characteristics of entrepreneurial entrepreneurs are explained in detail through relevant theories. Subsequently, the spiritual and psychological characteristics of entrepreneurs are expounded, and the influence and correlation of entrepreneurship and entrepreneurial psychological characteristics are analyzed on corporate social responsibility. Here, the aim is to provide scientific and effective references for follow-up research on entrepreneurship. The methods are scientific and the content is comprehensive. The specific role of entrepreneurship is innovatively analyzed from the perspective of marketization, which has some theoretical value and practical significance.

## Literature Review

### Definition and Characteristics of the Entrepreneurial Entrepreneur

Innovation is the essence of entrepreneurship, so entrepreneurs who promote and implement entrepreneurship play an important role and task in the venture creation process. Successful entrepreneurs need to have excellent psychological quality, an optimistic attitude toward life, self-confidence, and a strong desire for a career (Rogoza et al., 2018). The definitions of entrepreneurs are diverse in different disciplines. Economists believe that entrepreneurs can add value, create profits, or make appropriate innovations through mobilizing or utilizing resources. Psychologists think that entrepreneurs are those who have the right to avoid a particular outcome or achieve a particular purpose for a particular motivation (Buschow and Laugemann, 2020). All the above definitions of entrepreneurial entrepreneurs include characteristics, such as innovativeness, independence, and adventurousness. These can lay the theoretical basis for the study of the psychological characteristics of entrepreneurs. Here, entrepreneurship and psychological characteristics are specifically studied in entrepreneurial personal traits that include values, attitudes, personality traits, motivation, goals, selection tendencies, and other research elements.

### Entrepreneurship and Psychological Characteristics of Entrepreneurial Entrepreneurs

Entrepreneur's psychological characteristics greatly affect the development of enterprises, so the research on the psychological characteristics of entrepreneurs is of great significance. Character is an individual's personality that can be proved through an individual's behavior and the psychological process, containing various psychological characteristics. Meanwhile, an individual's characters are mostly shaped by heredity and social and natural environment (Chen, 2019). There are many related works on the psychological characteristics of entrepreneurs both in China and foreign countries. Scholar Wu believed that entrepreneurs' psychological characteristics included the willingness to control fate, competitors' plan implementers, moderate risk preference, and independent personality (Wu et al., 2019). The works of Alsati showed that entrepreneurs had enough knowledge to cope with market changes, adventure, bold innovation, scientific management skills, and willingness to cooperate, which were the unique qualities for successful entrepreneurship (Alsaaty, 2017). Listingrum thought that personal achievement factors, such as motivation, values, and background, will affect entrepreneurs (Listiningrum et al., 2020). Scholar Sunarni believed that non-entrepreneurs and entrepreneurs could be distinguished through three main factors: risk preference, high demand for achievement, and internal control points (Sunarni et al., 2018). Entrepreneurship is dynamic, influenced by the environment, and can adapt to the active and passive conditions of sustainable development. Audretsch suggested that the characteristics of individuals or companies at their early establishment were the focus of traditional entrepreneurial research (Audretsch et al., 2017). When advocating the concept of entrepreneurship, many scholars have studied different aspects of entrepreneurship. Trujillo-Barrera et al. (2016) recommended measuring whether entrepreneurship was included in business activities from three dimensions of innovation, initiative, and risk-bearing. Luca (2017) studied the above three dimensions of performance and discussed whether all aspects of entrepreneurship would affect performance. Fairhurst et al. (2020) measured the company's entrepreneurship by bringing innovation, risk preference, and aggression into its index system. Pellegrini and Ciappei (2015) said that entrepreneurship needed to be restructured on a moral basis.

### Corporate Social Responsibility

Several definitions have been put forward for corporate social responsibility in the related research field. Agudelo and Davídsdóttir (2019) suggested that corporate social responsibility should be a series of obligations for businessmen to make decisions and implement according to social development goals and mainstream values. Adnan et al. (2018) divided corporate social responsibility into four responsibilities: economic, legal, charitable, and free decision-making. Companies were economic units that profit from production and operations and contribute to social and economic development. Although many studies have been conducted on the consequences of corporate social responsibility, there are relatively few empirical studies on corporate social responsibility from the perspective of entrepreneurs. The findings of Anggadwita et al. (2017) showed that the new direction of corporate social responsibility research was the impact of entrepreneurs' psychological characteristics and values on corporate social responsibility. Tiba et al. (2019) pointed out that the future direction of the healthy and sustainable development of enterprises was to strengthen the exploration of the relationship between entrepreneurship and corporate social responsibility and play a complementary role between them.

When scholars define the concept of corporate social responsibility, they also focus on the content of corporate social responsibility and clarify the part that companies need to perform. Sila and Cek (2017) divided corporate social responsibility into six aspects: financial responsibility, environmental responsibility, consumer responsibility, employee responsibility, charitable responsibility, and legal responsibility. Abu Zayyad et al. (2021) divided corporate social responsibility into eight aspects: responsible governance, economic development, human rights, labor practices, environment, fair management, consumer issues, and community development.

Based on the above theoretical definitions and the actuality here, the psychological characteristics of Chinese entrepreneurial entrepreneurs are specifically analyzed from the perspective of marketization, corporate social responsibility, and stakeholders. Based on the factor analysis and structural equation method, a questionnaire survey (QS) is conducted for middle-level and senior managers in different regions. Then, another QS is issued for the empirical analysis of the relationship between corporate social responsibilities through both qualitative analysis and quantitative analysis.

### The Relationship Between Social Culture and Enterprise Development

Social culture is a vivid portrayal of regional development. The social culture affects the overall development of the region (Vuong et al., 2018, 2020). Social and cultural factors have always been the focus of entrepreneurship research. The relationship between culture and economy is also emphasized by researchers. Many studies have shown that cultural factors have played an important role in economic development. Firstly, the individual economy is greatly affected by social culture, and the awareness of the individual economy promotes the market economy. Secondly, entrepreneurship is impacted by social culture. Particularly, social culture greatly impacts entrepreneur group consciousness, thus resulting in many regional entrepreneur groups. Vuong (2016) showed that geographical location and social culture influenced the entrepreneurship of Vietnamese entrepreneurs. Consequently, under the influence of social culture, a regional development model with distinctive characteristics was proposed. Today, China's booming economy and accelerating marketization have brought both opportunities and challenges to enterprises. Therefore, the current social environment and the enterprise marketization background should be fully considered for relevant research on enterprise development (Hahn et al., 2020).

## Methodology

### Analytic Methods

#### Key Variables Measurements

The entrepreneurship and psychological characteristics are evaluated based on existing research literature and the specific situation of China. Here, relevant research scales are included in the measurement of entrepreneurship and psychological characteristics and are discussed from five dimensions, including the innovative spirit, venturous spirit, enterprising spirit, cooperation spirit, and world-saving spirit. The analysis is based on the following three aspects of understanding entrepreneurship: innovation, adventure, and enterprising. Meanwhile, cooperation spirit and world-saving spirit are also introduced into the entrepreneurship spirit. Consequently, entrepreneurship spirit encompasses 24 psychological characteristics scale and psychological characteristics scale items. The scale uses the five-point Likert method and is divided into five grades: very unqualified, unqualified, general, qualified, and very qualified. Each option will be assigned 1–5 scores.

The marketization process data are calculated based on the regional marketing index from China National Marketization Process Index Report (2018). In the research on corporate social responsibility, scholars tend to study corporate social responsibility from the perspective of stakeholders, but scholars continue to debate which stakeholders should bear corporate social responsibility. This study draws on the views of Chinese entrepreneurs in integrating the work of domestic and foreign scholars and fulfilling corporate social responsibility. The purpose is not to conduct a detailed study of specific issues, so a more reasonable stakeholder method is used for variable measurement. Finally, corporate social responsibilities are divided into environmental responsibility, employee responsibility, consumer responsibility, legal responsibility, and community responsibility. The scale uses the five-point Likert method, there are five graded options, and according to the degree of conformity, each option is assigned to 1-5 points.

#### Research Data Sources and Reliability and Validity Tests

To ensure the reliability and validity of the survey, first, some sample data are collected as a pre-survey and analyzed. Then, the scale of items is optimized for formal surveys. The test period is from June 2020 to July 2020. The subjects are middle-level and senior managers, such as the chairman, general manager, deputy general manager, and person in charge. The role of decision-making is discussed next. Three methods are chosen to collect QS data. 1. The XX University MBA Center is utilized to investigate MBA students in summer classes. 2. An on-site QS can communicate closely with the managers. 3. An online QS is also designed. The sample size has been enlarged to improve the reliability and validity of the QS. A total of 120 QSs are distributed in three ways, and 113 valid QSs are recovered, with an effective recovery rate of 94.17%.

The SPSS22.0 statistics software is chosen for reliability and validity test, mainly from three perspectives: entrepreneurship and psychological characteristics, marketization process, and corporate social responsibility. KMO and Approximate chi-square values are selected as the evaluation indexes.

### Theoretical Foundation

#### Theoretical Research Model

**Figure 1:**
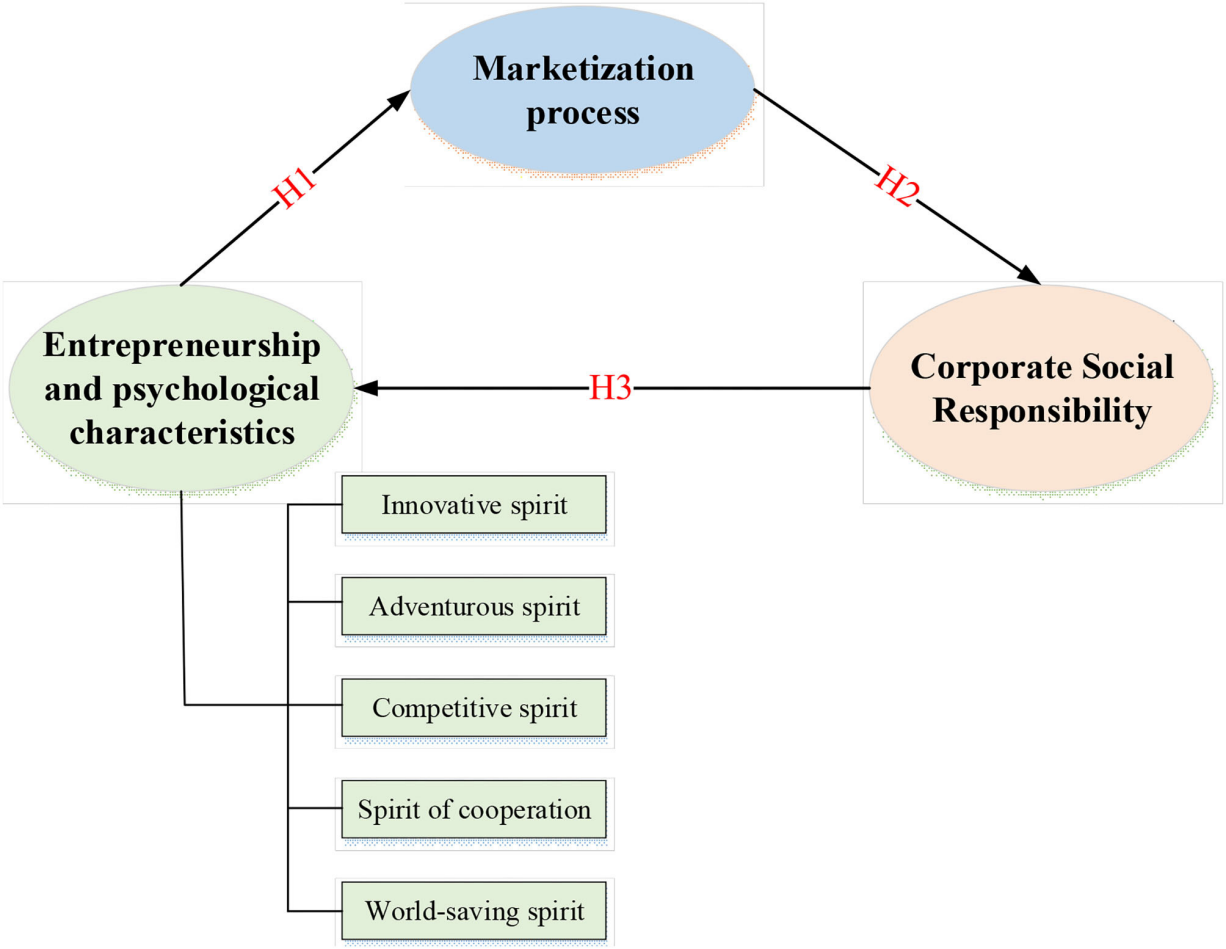
Relationship model of entrepreneurship and psychological characteristics, marketization process, and corporate social responsibility.

The impact of entrepreneurship and psychological characteristics on corporate social responsibilities is studied in detail through literature review and theoretical analysis from the perspective of marketization in current Chinese enterprises. Meanwhile, the verification mechanism is introduced for the marketization process. The mechanism of entrepreneurship, psychological characteristics, and corporate social responsibility are explored here, and their relationships are studied, providing a reference for modern enterprises to better fulfill their social responsibility. A theoretical model is established for the relationship among entrepreneurship and psychological characteristics, marketization process, and corporate social responsibility (Figure 1).

In the relationship model, entrepreneurship and psychological characteristics are divided into five dimensions: an innovative spirit, adventurous spirit, enterprising spirit, cooperation spirit, and world-saving spirit. The marketization process is a single dimension, and corporate social responsibility is a single dimension. The relationship between the research variables included in the theoretical model is as follows. First, entrepreneurship and psychological characteristics significantly and positively impact corporate social responsibility. Second, the marketization process also positively affects corporate social responsibility. Third, the process of marketization is the intermediary variable of entrepreneurship and psychological characteristics, and corporate social responsibility.

#### Research Hypothesis

Based on the above research theories and specific requirements, the corresponding hypotheses are designed below.

Hypothesis 1: entrepreneurship has a great positive impact on corporate social responsibility.

H1a: innovative spirit has a great positive impact on corporate social responsibility.

H1b: adventurous spirit has a great positive influence on corporate social responsibility.

H1c: the enterprising spirit has a great positive influence on corporate social responsibility

H1d: the cooperation spirit has a great positive impact on corporate social responsibility.

H1e: the world-saving spirit has a great positive influence on corporate social responsibility

H2: there is a significant positive correlation between the marketization process and corporate social responsibility. The faster the marketization process of the company is, the better the corporate social responsibility performance is.

H3: the marketization process plays an important and positive intermediary role in the relationship between entrepreneurship and corporate social responsibility. The faster the marketization process of the company is, the stronger the role of entrepreneurship in promoting corporate social responsibility is.

## Results

### Test Results of Reliability and Validity of the Scale

**Figure 2:**
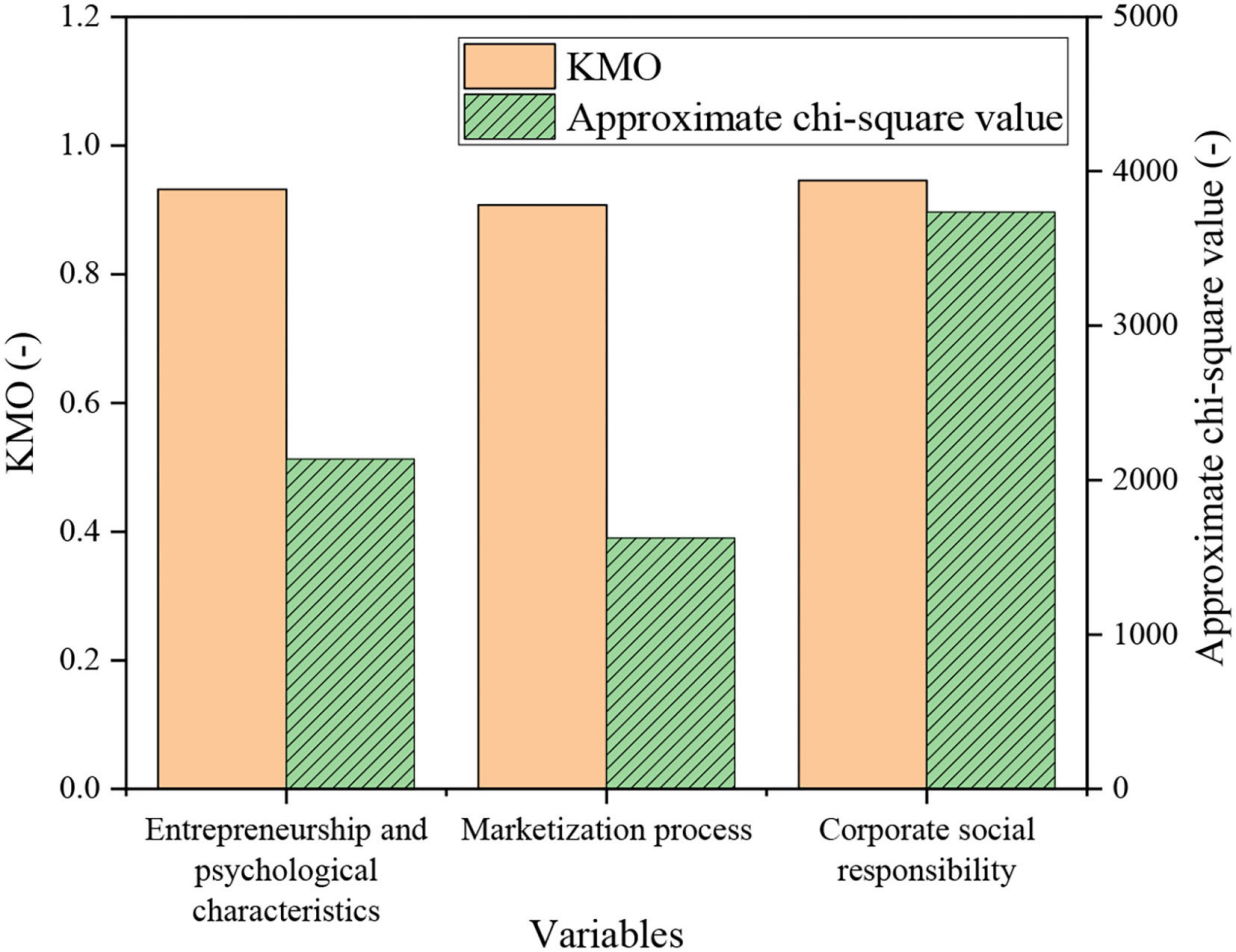
Test results of reliability and validity of the scale.

Figure 2 shows the specific test results of reliability and validity.

The alpha values of innovative spirit, adventurous spirit, enterprising spirit, cooperation spirit, and world-saving spirit are 0.862, 0.843, 0.864, 0.768, and 0.873, respectively, all >0.7. It suggests that the entire entrepreneurship and psychological characteristics scale has excellent reliability. Three KMO (Kaiser-Meyer-OIkin) values and Bartlett fields are introduced next. The KMO values of the three variables are >0.9. In the meanwhile, the approximate chi-square value of the Butterlit ball test is significant (sig = 0.000). Thus, the scale has good reliability and validity and can be utilized for the experiment.

### Results of Key Variables Analysis

Here, the SPSS can analyze the relationship between variables in the relationship model. Correlation includes positive and negative correlations. The premise of regression analysis is that there is a certain difference in correlation between variables. Pearson's multiplicative-product difference correlation method can analyze the relationship among three key variables. The specific analysis results are shown in Table 1.

**Table 1:** Results of key variables analysis and their dimensions.

**Variables**	**1**	**2**	**3**	**4**	**5**	**6**	**7**	**8**
1. Entrepreneurship	1							
2. Innovative spirit	0.846^**^	1						
3. Adventurous spirit	0.745^**^	0.573^**^	1					
4. Enterprising spirit	0.813^**^	0.594^**^	0.526^**^	1				
5. Cooperation spirit	0.795^**^	0.603^**^	0.427^**^	0.612^**^	1			
6. World-saving spirit	0.852^**^	0.613^**^	0.576^**^	0.605^**^	0.631^**^	1		
7. Marketization process	0.776^**^	0.625^**^	0.479^**^	0.638^**^	0.654^**^	0.896^**^	1	
8. Corporate social responsibility	0.868^**^	0.693^**^	0.533^**^	0.743^**^	0.768^**^	0.792^**^	0.854^**^	1

** * Represents a significant correlation at 0.01 level*.

Table 1 displays that the coefficient of the marketization process to entrepreneurship is 0.776, which is significantly correlated at the level of 0.01. Of the five dimensions of entrepreneurship, the coefficient of world-saving spirit to marketization process is the highest, which is 0.896; the coefficient of adventurous spirit to marketization process is the lowest, which is 0.479; and the coefficients of all dimensions to marketization process are significant at the level of 0. 01. The coefficient of entrepreneurship spirit to corporate social responsibility is 0.868, which is also significant at the level of 0.01; specifically, of the five dimensions of entrepreneurship, the coefficient of world-saving spirit to corporate social responsibility is the highest, which is 0.792; and the coefficient of the adventurous spirit is the lowest, which is 0.533.

### Regression Analysis of Entrepreneurship and Psychological Characteristics on Corporate Social Responsibility

Regression analysis can explore the influence of each variable in the model based on correlation analysis, and the main effect regression analysis is completed in two steps. First, entrepreneurship and psychological characteristics are set as independent variables, while corporate social responsibility is set as the dependent variable and analyzed through regression analysis. Second, the innovative spirit, adventurous spirit, enterprising spirit, cooperation spirit, and world-saving spirit are set as independent variables, while corporate social responsibility set as the dependent variable and analyzed through regression analysis. Table 2 shows the regression analysis results of entrepreneurship and its dimensions on corporate social responsibility.

**Table 2:** Regression analysis of entrepreneurship and psychological characteristics on corporate social responsibility.

**Variables**	**Corporate social responsibility**		
**Control variable**	**Model 1**	**Model 2**	**Model 3**
Gender	−0.09	0.067	
Age	−0.053	0.054	
Academic credential	−0.184^*^	0.046	
Working years	0.216	0.046	
**Independent variable**			
Entrepreneurship and psychological characteristics		0.863^***^	
Innovative spirit			0.174^**^
Adventurous spirit			−0.045
Enterprising spirit			0.263^***^
Cooperation spirit			0.287^***^
World-saving spirit			0.502^***^
*F* value	3.258^***^	95.932^***^	63.647^***^
*R* ^2^	0.064	0.742	0.812
Δ*R*^2^	0.041	0.731	0.805
Dw		2.05	2.14

* * Represents p <0.05*,

** *denotes p <0.01, and*

*** * stands for p <0.001*.

To verify hypothesis 1, the regression analysis can be divided into two steps, with entrepreneurship set as the independent variable and corporate social responsibility set as the dependent variable. Step 1: construct Model 1 by taking the four demographic variables of the subjects as the control variables and putting them into the regression equation with corporate social responsibility as the dependent variable. Step 2: introduce entrepreneurship into Model 1, and form Model 2. In the process of testing Model 1 and Model 2, the DW value of the regression model is 2.05, indicating that there is no autocorrelation between items. Compared with Model 1, the F value of Model 2 is significant at the levels of 0.001 and Δ*R*^2^ = 0.742, indicating that the fitting effect of Model 2 is better than that of Model 1. The β value of entrepreneurship on corporate social responsibility is 0.863 (*p* < 0.001), indicating that entrepreneurship apparently and positively influences corporate social responsibility. Thus, hypothesis H1 is verified.

Entrepreneurship is regarded as a high-order variable and can influence corporate social responsibility from five aspects: an innovative spirit, adventurous spirit, enterprising spirit, cooperation spirit, and world-saving spirit. The first step is to build model 1, the second step is to build model 3 based on model 1, adding five independent variables: an innovative spirit, adventurous spirit, enterprising spirit, cooperation spirit, and the world-saving spirit. The DW value of the regression model is 2.05 and there is no autocorrelation. Compared with Model 1, Model 3 has a significant F value, Δ*R*^2^ = 0.812, indicating that Model 3 has a better fitting effect than Model 1. The β values of innovative spirit, adventurous spirit, enterprising spirit, cooperation spirit, and corporate social responsibility are 0.174 (*p* < 0.01), −0.045, 0.263 (*p* < 0.001), 0.287 (*p* < 0.001), and 0.502 (*p* < 0.001), respectively. It reveals that innovative spirit, enterprising spirit, cooperation spirit, and corporate social responsibility have a significant positive impact. The adventurous spirit will not have a significant impact on corporate social responsibility. Hypotheses H1a, H1c, H1d, and H1e are verified to be valid, but hypothesis H1b is tested as invalid.

### The Multiple Regression Analysis of the Relationship Between Marketization Process and Corporate Social Responsibility

The relationship between the marketization process and corporate social responsibility is analyzed to verify hypothesis 2 based on the multiple regression analysis methods, and the regression equation is obtained (Table 3).

**Table 3:** Marketization process and corporate social responsibility regression analysis results.

**Variable**	**Unstandardized**		**Standardized system Beta**	**t**	**Significance**	***R*^**2**^**	**Δ*R*^**2**^**	***F* value**	**Significance**
	**B**	**Standard error**							
Constant	−38.563	6.954		−5.362	0.000	0.185	0.174	31.025	0.000
MI	0.537	0.176	0.173	3.637	0.000				
Company size	3.763	0.295	0.302	9.462	0.000				
Profit capacity	0.785	0.468	0.072	1.637	0.068				

Table 3 demonstrates that the adjusted R2 of the model is 0.174, and the fitting degree of the model is acceptable. The F value is 30.904, and the significance is 0.000, indicating that the model has passed the significance test. The regression results show that the regression coefficient of the marketization process (model 1) is 0.574, and the significance is <0.01. In other words, the marketization process and corporate social responsibility are at 1%. Thus, hypothesis H2 is true. The faster the marketization process of the company's market environment is, the more positive the corporate social responsibility is. In the control variables, only the company size has passed the significant test, and the relationship between profitability and corporate social responsibility is not significant.

### Analysis of the Mediating Effect of Marketization

To test the intermediary effect of the marketization process on the relationship between entrepreneurship and psychological characteristics and corporate social responsibility, the marketization process is introduced into the regression equation of entrepreneurship and corporate social responsibility for analysis. The results are shown in Table 4.

**Table 4:** The results of regression analysis of the intermediary effect of marketization.

**Variables**	**Corporate social responsibility**		
**Control variable**	**Model 1**	**Model 2**	**Model 4**
Gender	−0.09	0.067	
Age	−0.053	0.054	
Academic credential	−0.184^*^	0.046	
Working years	0.216	0.046	
**Independent variable**			
Entrepreneurship and psychological characteristics		0.863^***^	0.524^***^
Intermediate variable			
Marketization process			0.502^***^
*F* value	3.258^***^	95.932^***^	87.537^***^
*R* ^2^	0.064	0.742	0.843
Δ*R*^2^	0.041	0.731	0.812
Dw		2.05	2.09

* *Represent p <0.05 and*

*** * stands for p <0.001*.

Table 4 indicates that the marketization process is introduced as a comprehensive variable into the regression equation of entrepreneurship and corporate social responsibility, as shown in Model 4. The results show that the DW value of this model is 2.09, close to 2, indicating that this sequence has no autocorrelation. Entrepreneurship and psychological characteristics have a significant positive impact on corporate social responsibility (M2, β = 0.863, *p* < 0.001), which can explain all 74.2% of the changes in corporate social responsibility. When the market process is introduced, entrepreneurship has a great positive impact on corporate social responsibility (M4, β = 0.524, *p* < 0.001), and the market process has a great positive impact on corporate social responsibility. While the impact of entrepreneurship and psychological characteristics on corporate social responsibility has decreased (from 0.863 to 0.524). This shows that the marketization process plays an intermediary role between entrepreneurship and corporate social responsibility. Thus, hypothesis H3 is true. To sum up, the specific results of the research hypotheses are summarized in Table 5.

**Table 5:** Test results of the proposed hypothesis.

**Serial number**	**Hypothesis**	**Results**
H1	Entrepreneurship has a great positive impact on corporate social responsibility.	True
H1a	Innovative spirit has a great positive influence on corporate social responsibility.	True
H1b	The adventurous spirit has a great positive influence on corporate social responsibility.	False
H1c	Enterprising spirit has a great positive impact on corporate social responsibility.	True
H1d	Cooperation spirit has a great positive impact on corporate social responsibility.	True
H1e	The world-saving spirit has a great positive impact on corporate social responsibility.	True
H2	There is a significant positive correlation between the process of marketization and corporate social responsibility. The faster the marketization process of the company is, the better its corporate social responsibility performance is.	True
H3	The marketization process plays an important positive intermediary role in the relationship between entrepreneurship and corporate social responsibility. The faster the marketization process of the company is, the stronger the role of entrepreneurship in promoting corporate social responsibility is.	True

## Discussion

The results of variable analysis between the relational models show that the dimensions of entrepreneurship and psychological characteristics are significantly and positively correlated with corporate social responsibility. The dimensions of entrepreneurship are significantly positively correlated with the marketization process, and the marketization process is also positively correlated with corporate social responsibility significantly. Specifically, based on the research data, there is a positive correlation between the innovation spirit and the marketization process, and combined with the actual situation, the innovation spirit has promoted social development and the marketization process. There is also a correlation between corporate social responsibility and the marketization process, and corporate social responsibility has a positive impact on the marketization process.

The hypothesis test results of regression analysis reflect that the senior managers of enterprises are researched, the relationship is discussed among entrepreneurship and psychological characteristics, marketization process, and corporate social responsibility through empirical research method. Hence, entrepreneurship can directly and positively affect corporate social responsibility and indirectly and positively affects corporate social responsibility through the intermediary effect of the marketization process.

## Conclusion

Here, the impact of entrepreneurship and psychological characteristics on social responsibility is mainly analyzed in the marketization process. The main conclusions can be summarized as follows. 1. The positive impact of entrepreneurship on corporate social responsibility is significant. 2. Innovative spirit, adventurous spirit, enterprising spirit, cooperation spirit, and world-saving spirit have a significant positive impact on corporate social responsibility. 3. Adventurous spirit has no significant impact on corporate social responsibility. 4. There is a significant positive correlation between the marketization process and corporate social responsibility, that is, the faster the marketization process in the location of the enterprise is, the better the performance of corporate social responsibility is. Here, the intermediary role of the marketization process is verified for the relationship between entrepreneurship and corporate social responsibility. The marketization process has a significant positive intermediary effect on the relationship between entrepreneurship and corporate social responsibility, that is, the faster the marketization process is, the stronger the role of entrepreneurs in promoting corporate social responsibility is. The theoretical analysis and case study are combined, and the research model is established to scientifically analyze the correlation among entrepreneurship, corporate social responsibility, and the marketization process. The research method is scientific, and the results have some application value. However, due to time and costs limitations, the surveyed subjects are only chosen from one class, so the size and the scope of samples are relatively small. In the follow-up study, the scope and size will be expanded to multiple regions and diverse backgrounds, and the practicability of the research results will be strengthened.

## Data Availability Statement

The raw data supporting the conclusions of this article will be made available by the authors, without undue reservation.

## Ethics Statement

The studies involving human participants were reviewed and approved by Huaiyin Normal University Ethics Committee. The patients/participants provided their written informed consent to participate in this study. Written informed consent was obtained from the individual(s) for the publication of any potentially identifiable images or data included in this article.

## Author Contributions

All authors listed have made a substantial, direct and intellectual contribution to the work, and approved it for publication.

## Conflict of Interest

The authors declare that the research was conducted in the absence of any commercial or financial relationships that could be construed as a potential conflict of interest.

## Publisher's Note

All claims expressed in this article are solely those of the authors and do not necessarily represent those of their affiliated organizations, or those of the publisher, the editors and the reviewers. Any product that may be evaluated in this article, or claim that may be made by its manufacturer, is not guaranteed or endorsed by the publisher.
